# xHD-Vox, an Automated Speech Model for Estimating Motor and Cognitive Scores in Huntington Disease: Development and Longitudinal Validation

**DOI:** 10.2196/83838

**Published:** 2026-07-08

**Authors:** Tiphaine Le Ludec, Andres Gil-Salcedo, Hadrien Titeux, Robin Louiset, Clément Le Moine Veillon, Renaud Massart, Anne-Catherine Bachoud-Lévi

**Affiliations:** 1 Team 01 - Neuropsychologie Interventionnelle, INSERM U955, Mondor Institute of Biomedical Research, Créteil, France; 2 NeurATRIS, Créteil, France; 3 Department of Cognitive Science, Ecole Normale Supérieure (ENS), 29 rue d'Ulm, Paris, 75005, France, 33 680728417; 4 Reference Center for Huntington’s Disease, Department of Neurology, Henri Mondor-Albert Chenevier University Hospitals, Créteil, France; 5 Faculty of Medicine, Université Paris-Est Créteil, Créteil, France

**Keywords:** huntington, speech, voice, automated, cUHDRS, UHDRS, machine learning, longitudinal, telemonitoring, motor and cognitive function, composite Unified Huntington Disease Rating Scale, Unified Huntington Disease Rating Scale

## Abstract

**Background:**

Huntington disease (HD) is a rare genetic neurodegenerative disease that causes progressive motor, cognitive, and psychiatric symptoms over decades after onset. Clinical care is typically provided in specialized centers with only annual clinical assessments, highlighting the need for more frequent and cost-effective monitoring.

**Objective:**

This study aimed to develop and validate xHD-Vox, a fully automated, interpretable, speech-based model for predicting the composite Unified Huntington Disease Rating Scale (cUHDRS) and its cognitive, motor, and functional components.

**Methods:**

We included 181 HD gene carriers (341 annual visits) from three French prospective cohorts: BIO-HD (NCT01412125), REPAIR-HD (NCT03119246), and MIG-HD (NCT00190450). Participants had ≥40 cytosine-adenine-guanine (CAG) repeats, available cUHDRS scores, and audio recordings of forward and backward counting (1-20). For model development and feature selection, we used a speech pathologist-annotated subset (145 visits and 90 participants). Selected speech features were then automated using Whisper, an open-source speech recognition tool. The final linear regression model, xHD-Vox, was calibrated on the training set of 269 visits (157 participants, and annotated subset included). Performance was evaluated on an independent longitudinal test set (24 participants, with 3 annual visits each) using mean absolute error, explained variance (*R*^²^), and intraclass correlation coefficient. Longitudinal decline was assessed with 2-way repeated-measures ANOVAs. Predicted 1-year and 2-year changes were compared with clinician-assessed 95% CIs.

**Results:**

Feature selection identified four key predictors: standardized CAG-age-product score, CAG repeat length, rate of numbers pronounced per second, and the SD of that rate. On the test set, xHD-Vox achieved a mean absolute error of 2.1 for cUHDRS and explained 57% of its variance, compared with 38% when using only demographic features. Longitudinal analyses using repeated-measures ANOVAs with post hoc Tukey tests confirmed a significant decline over the 2-year follow-up for both clinician-assessed measures and xHD-Vox predictions. At the group level, the mean 1-year and 2-year changes predicted by xHD-Vox were consistent with clinically measured changes, falling within the corresponding 95% CIs.

**Conclusions:**

We developed xHD-Vox, an interpretable and automated model that predicts clinical scores in HD using a short speech task. Predicted scores were consistent with clinician-assessed scores, supporting its potential use in mobile apps for remote monitoring. This approach could facilitate scalable, real-time tracking of disease progression, especially in underserved regions, and enable personalized and responsive clinical care.

## Introduction

Huntington disease (HD) is a rare autosomal neurodegenerative disorder caused by an expanded cytosine-adenine-guanine (CAG) repeat (>35) in the *huntingtin* gene [1]. The disease manifests through a variety of motor, cognitive, and psychiatric symptoms, requiring lifelong multidisciplinary care. While no cure is currently available despite extensive research on disease modifiers, symptomatic treatments are effective in managing disability for many years. Therefore, continuous and personalized monitoring of disease progression is essential to optimize patient care. However, access to specialized expertise remains limited, highlighting the need for scalable, automated approaches to support clinical decision-making.

The composite Unified Huntington disease Rating Scale (cUHDRS) is a robust metric for tracking disease progression, integrating motor, cognitive, and functional scores from the UHDRS [2]—the worldwide reference scale for HD clinical evaluation. Its assessment is typically conducted once a year during face-to-face examinations by trained neurologists and neuropsychologists with restricted availability [3]. This calls for cost-effective and remote-friendly solutions to enable continuous disease monitoring.

Speech analysis has emerged as a promising, noninvasive, and practical tool for tracking HD progression [4,5]. While other biomarkers, such as striatal atrophy on magnetic resonance imaging, cerebrospinal fluid neurofilament levels, and cognitive batteries, have been validated to monitor disease progression, speech-based assessments are well-suited for remote, home-based implementation, reducing the logistical burden and costs associated with in-clinic visits. In our previous study [5], we demonstrated that speech features could predict cUHDRS scores and their cognitive and motor components more accurately than the combination of demographic and genetic data [5]. These features were extracted from a simple counting task, in which participants counted forward and backward from 1 to 20 in less than 45 seconds. However, the need for manual annotations by speech pathologists still limited the model’s scalability and implementation as a telemonitoring solution [6,7].

To address these limitations, we developed xHD-Vox (x for eXplainable, HD for Huntington disease, and Vox for voice), an interpretable and automated model that predicts cUHDRS and its motor and cognitive components from brief speech recordings in HD gene carriers. This study comprises the following two main parts: (1) xHD-Vox development, where we ensured interpretability by using a standard linear regression model and selecting a subset of relevant speech features identified in our previous work [5]. Manual annotations were replaced with features extracted using Whisper, an open-source automatic speech recognition (ASR) system developed by OpenAI, known for its high transcription accuracy and multilingual support [8]. (2) xHD-Vox evaluation on an independent longitudinal dataset to evaluate its ability to track disease progression over time.

## Methods

### Participants

Participants were enrolled from three prospective longitudinal studies: BIO-HD (NCT01412125), REPAIR-HD (NCT03119246), and MIG-HD (NCT00190450) before transplants.

**Figure 1.**
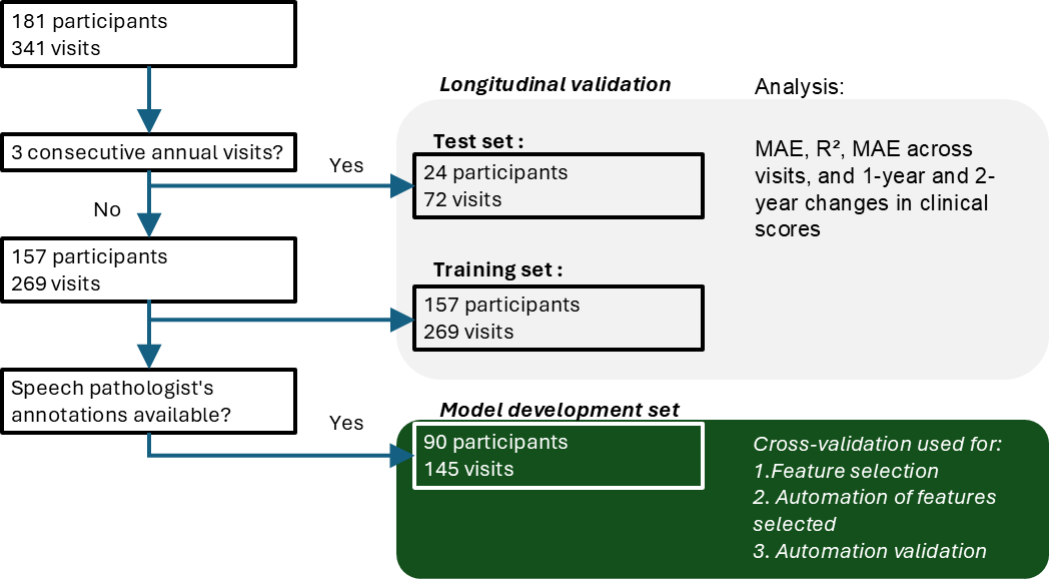
Participant selection flowchart for the development and validation study. The test set for validation is first isolated from the database. Model development is performed on a subset of the training set with speech pathologists’ annotations available. MAE: mean absolute error; *R*^2^: explained variance.

We selected data from HD gene carriers with a CAG repeat length of greater than or equal to 40 to ensure the complete penetrance of HD [9]. We excluded visits with missing speech recordings or clinical scores required to compute cUHDRS. This resulted in the selection of 181 participants, with a total of 341 visits (Figure 1).

For the evaluation of our model on an independent longitudinal test set, we selected participants who had 3 consecutive annual visits, which resulted in a cohort of 24 participants (72 visits). Assessing performance over a 2-year follow-up period ensured a sufficiently long observation window to capture disease progression.

The remaining 157 participants, comprising 269 visits (mean 1.7, SD 1.1), were used for model training.

Among these, 145 recordings from 90 participants were annotated by speech pathologists and used to develop the automated model.

### Clinical Evaluation

Participants were assessed by annually certified examiners with the Unified Huntington Disease Rating Scale (UHDRS). For this study, we used the cUHDRS and its four components to evaluate the disease progression: Total Motor Score (TMS), the Symbol Digit Modalities Test (SDMT), the Stroop Word (SW), and the Total Functional Capacity (TFC). The cUHDRS was calculated using the following formula [2]:


$$\begin{array}{rl} cUHDRS & =\frac{TFC-10.4}{1.9}-\frac{TMS-29.7}{14.9}\\ \;+\frac{SDMT-28.4}{11.3}+\frac{SW-66.1}{20.1}+10 \end{array}$$

The CAG-age-product (CAP) score was calculated using the standardized formula calibrated to reach 100 at the expected age of onset [10]:


$$CAP=age\times \frac{CAG-30}{6.49}$$

### Standardized Lightweight Speech Task

Speech samples were recorded in a clinical research environment at Henri Mondor Hospital through 2 brief controlled tasks. Each participant was asked by a neuropsychologist to (1) count aloud numbers from 1 to 20 (forward counting), then (2) to count backward from 20 to 1 while holding their hands up and closing their eyes (backward counting). The backward task increases cognitive load by inhibiting the forward counting process [5]. Recordings were made using an external microphone (Zoom H4n Pro; 44 kHz recording, resampled at 16 kHz) for the BIO-HD and REPAIR-HD cohorts and were extracted from the audio track of video recordings for MIG-HD. The microphone was placed on the neuropsychologist’s desk, facing the patient, who was seated approximately 1‐2 meters away.

For the annotated samples, speech recordings were transcribed by speech therapists at the word level using the Praat (developed by Paul Boersma, David Weenink, and Anastasia Shchupak) and Seshat (developed by Hadrien Titeux, Rachid Riad, Xuan-Nga Cao, Nicolas Hamilakis, Kris Madden, Alejandrina Cristia, Anne-Catherine Bachoud-Lévi, and Emmanuel Dupoux) platforms [11]. Mispronunciations (using the Speech Assessment Methods Phonetic Alphabet), paraphasias, phoneme perseverations, abnormal breathing, vocal noises, and filled pauses (“euh” and ”um”) were identified.

### Analysis Implementation

This study followed the TRIPOD+AI (Transparent Report of a Multivariable Prediction Model for Individual Prognosis or Diagnosis) reporting guideline. We conducted the analysis in Python using the scikit-learn library [12]. Features were normalized with RobustScaler to enhance feature importance analysis and improve the model’s convergence. For cross-validation, we generated 50 folds, with each fold containing 80% of participants in the training set and the remaining 20% in the test set. Each participant was assigned exclusively to either the training or test set within each fold.

### Automated Model Development

#### Overview

In this first analysis, we automated the model developed by Riad et al [5] for predicting cUHDRS and its components using a parsimonious approach to reduce model complexity while preserving predictive accuracy. This approach involved the following three steps: (1) ranking the most predictive features of Riad et al [5] model, (2) identifying the smallest subset of features that achieved at least the same performance as the full feature set by progressively adding features in order of importance, and (3) automating the annotation process for the selected features using Whisper.

#### Ranking the Most Predictive Features of our Previous Model

##### Features Investigated

We applied the automatic pipeline for feature extraction based on speech pathologists’ annotations previously developed by Riad et al [5]. This process resulted in the extraction of 60 speech features (30 features for each of the 2 tasks), including articulatory and phonatory deficiencies, rhythm and temporal statistics, filled pauses, vocalization additions, and sequence-related metrics (eg, errors in number order and perseveration errors) (Table S1 in Multimedia Appendix 1 provides the detailed list of features extracted). We excluded the feature phones per second due to their high correlation (r=0.99) with pronounced numbers per second*.* Demographic features, including CAP score, age, and CAG repeat length, were added, leading to a total of 63 features being investigated.

##### Features Importance Ranking

Riad et al [5] model is a 63-feature ElasticNet regression, a standard linear regression with L1 and L2 regularization to prevent overfitting and mitigate multicollinearity. The study demonstrated that using only features from the backward task yields performance comparable to that achieved using features from both forward and backward tasks (ablation study). Performance was assessed using mean absolute error (MAE) across 50 cross-validation folds, which quantifies the average absolute difference between observed and predicted clinical scores.

Given similar results in our replication (Tables S2 and S3 in Multimedia Appendix 1), we retained only features from the backward counting task for further selection.

To identify the most relevant features from this task, we calculated the average absolute value of the ElasticNet regression coefficients across 50 cross-validation training folds. Features were then ranked by their relative importance for each clinical score prediction. Finally, we computed an average rank for each feature across cUHDRS, TMS, and cognitive scores. TFC was excluded from this average ranking because the model showed low explained variance (*R*²) for this score, indicating poor predictive performance.

### Identifying the Minimal Predictive Features Set

To identify the minimal set of features that achieved performance at least equivalent to that of the initial model [5], features were progressively added in order of importance, as determined in step (1), in a standard linear regression model. We stopped adding features once the model’s performance matched or exceeded that of the initial model. Performance was evaluated using the mean MAE across 50 cross-validation folds. This method identified a minimal subset; however, it may not be globally optimal, as other combinations of features could potentially provide better predictive performance.

### Automating the Selected Features’ Annotation Process Using Whisper

The two selected features in step (2) were “numbers pronounced per second” and “SD of the duration of individual numbers.” Numbers pronounced per second were computed as the total number of numbers spoken (including perseverations and sequence errors) divided by the task duration. Task duration was defined as the time interval from the start of the first number pronounced to the end of the last number pronounced. The SD of duration was calculated across the duration of individual spoken numbers.

To automate the extraction of these 2 features, it was necessary to identify the sequence of numbers produced by each participant within recordings that could include hesitations, fillers, or unrelated speech (eg, questions addressed to the neuropsychologist). This required accurate transcription of the recordings as well as precise text-audio alignment to compute timing-related features.

We used Whisper, a pretrained ASR model developed by OpenAI [8], which provides both transcription and timestamp alignment, thereby facilitating automated extraction of timing features. Whisper was trained on 680,000 hours of labeled audio, including multilingual datasets, and has demonstrated a low word error rate in French (word error rate=5.3 on FLEURS and 10.8 on Common Voice [version 15]). Its robustness to noisy recordings and coverage of 96 languages made it well suited for this task.

Numbers pronounced per second and the SD of the duration of individual numbers were compared using Pearson and Spearman correlations between the values computed from Whisper’s annotations and those derived from speech-language pathologist annotations.

Finally, we validated our automated model by comparing the MAE of predictions based on features extracted from speech pathologists’ annotations with those obtained using Whisper’s alignment.

### Model Evaluation Using Longitudinal Data

#### Overview

We evaluated our automated model on longitudinal data using a leave-one-out setup to simulate its real-world performance. The model was calibrated on the training set before being tested on unknown participants (test set).

All longitudinal assessment results are reported on the test set to ensure evaluation on an unseen cohort and avoid inflated performance estimates. Given the test set selection approach, we verified a posteriori that (1) the training set contained enough participants for proper model calibration, (2) the training/testing split followed an 80/20 ratio in terms of visits, and (3) the test and training sets were balanced in terms of disease severity (refer to the Participants subheading in the Results section for details).

#### Model Performance on the Test Set

The model was trained using linear regression, and its performance was evaluated on the test set using the MAE, *R*², and intraclass correlation coefficient (ICC). The *R*² metric quantifies the proportion of variance explained by the model, whereas the ICC measures the agreement between predicted and observed clinical scores. To highlight the potential contribution of speech features, we compared the performance of our model with that of a baseline demographic model (based on CAP score and CAG repeat length).

#### Functional Classification

Given the low performance of the regression model for TFC, we explored functional decline using a logistic regression model trained to classify stage 3 vs stages 0‐2 of the Huntington Disease Integrated Staging System (HD-ISS). The model included two predictors: CAP score and speech rate (measured as numbers pronounced per second). Selection of these predictors was supported by cross-validation results (Figure S3 and methods in Multimedia Appendix 1). Model performance was evaluated using Receiver Operating Characteristic–Area Under the Curve (ROC AUC), accuracy, and recall.

#### Longitudinal Change

To test the hypothesis of a longitudinal decline in clinical scores, both as measured by clinicians and as predicted by the model on the test set, we performed 2-way repeated-measures ANOVAs separately for each score. The 2 within-subject factors were visit time (baseline, year 1, and year 2) and type of measure (clinician-assessed vs model-predicted). Statistical significance was set at *α*=.05, and Bonferroni corrected for the 5 tested scores.

For each ANOVA, we examined the main effects of time and type of measure, as well as their interaction. When a significant effect of time was observed, Tukey post hoc comparisons were conducted to characterize differences between visits.

To quantify the magnitude of longitudinal change, we computed 1-year and 2-year changes in each score for both clinician-assessed and model-predicted measures. One-year change was defined as the difference between the score at year 1 and baseline, whereas 2-year change was defined as the difference between the score at year 2 and baseline. Group-level agreement between clinical and predicted changes was assessed by comparing mean changes and their 95% CIs.

To evaluate model performance at the individual level, we examined the relationship between predicted and clinically measured 1-year changes using scatterplots, Pearson correlation coefficients, and Spearman correlation coefficients. This analysis was restricted to 1-year changes to maximize the number of observations (48 paired changes), thereby increasing statistical power. Sensitivity to outliers was assessed and reported in Multimedia Appendix 1.

### Ethical Considerations

All participants signed an informed consent. Ethical approval was obtained from the institutional review board of Henri Mondor Hospital for BIO-HD and MIG-HD and of Saint-Louis Hospital for the French part of REPAIR-HD. The study complied with the Declaration of Helsinki, current Good Clinical Practice guidelines, and local laws and regulations.

## Results

### Participants

The full database (N=181 participants, 341 visits) included individuals at early stages of the disease, with low to moderate functional impairment (mean TFC 11.2, SD 2.3). Detailed demographic and clinical characteristics are provided in Table 1.

This database was split into training and testing sets (refer to Figure 1 for the flow of subset construction). The test set comprised 21% of the total visits and was balanced in terms of disease severity compared with the training set: 50% of participants were at stage 3 in both sets, and 19% were at stage 0‐1 (vs 32% in the training set). Clinical scores (cUHDRS, TFC, SDMT, SW, and TMS) were also close between the 2 subsets.

Our sample size analysis (Figure S2 in Multimedia Appendix 1) indicated that 100 participants were sufficient to calibrate the model with 95% accuracy; this condition was met with 157 participants in the training set.

The speech pathologist-annotated database used for model development showed a disease severity profile similar to that of the full database, with a slightly lower mean TFC (mean 10.8, SD 2.3).

A PRISMA (Preferred Reporting Items for Systematic Reviews and Meta-Analyses) flow diagram of data selection is provided in Figure S1 in Multimedia Appendix 1. Only 1.2% of clinical data were missing.

**Table 1.** Demographic and clinical characteristics across all database subsets used in the different analyses.

Characteristic	Full database	Test set	Training set	Speech pathologist–annotated database
Number of participants	181	24	157	90
Sex, n				
Female	100	7	93	55
Male	81	17	64	35
Number of visits	341	72	269	145
Number of visits, mean (SD)	1.9 (1.1)	3 (0)	1.7 (1.1)	1.6 (0.8)
Age at first visit, mean (SD)	49.4 (11.3)	53.5 (10)	48.7 (11.4)	51.7 (10.5)
CAG^a^ repetition, mean (SD)	43.6 (2.9)	42.5 (1.7)	43.7 (3)	43.6 (3.4)
HD-ISS^b^ 0‐1, n visits (%)	100 (0.29)	14 (0.19)	86 (0.32)	37 (0.25)
HD-ISS 2, n visits (%)	73 (0.22)	22 (0.3)	51 (0.19)	27 (0.19)
HD-ISS 3, n visits (%)	168 (0.49)	36 (0.5)	132 (0.49)	81 (0.56)
cUHDRS^c^, mean (SD)	12.1 (4.6)	12.0 (4.1)	12.1 (4.8)	11.2 (4.6)
TMS^d^, mean (SD)	21.0 (18.8)	21.3 (16.5)	20.9 (19.4)	25.2 (19)
TFC^e^, mean (SD)	11.2 (2.3)	11.4 (1.9)	11.1 (2.4)	10.8 (2.3)
SDMT^f^, mean (SD)	35.7 (16.3)	34.4 (14)	36 (16.9)	32.7 (16.3)
SW^g^, mean (SD)	74.9 (23.7)	73.4 (21.8)	75.2 (24.1)	71.1 (23.6)

a CAG: cytosine-adenine-guanine.

b HD-ISS: Huntington Disease Integrated Staging System.

c cUHDRS: composite Unified Huntington Disease Rating Scale.

d TMS: Total Motor Score.

e TFC: Total Functional Capacity.

f SDMT: Symbol Digit Modalities Test.

g SW: Stroop Word.

### Automated Model Development

#### Ranking the Most Predictive Features

Table S3 in Multimedia Appendix 1 shows that the *R*² for the TFC score was low (0.13) compared with that of the other scores, which were closer to 0.5. Although we continued to report results for TFC, it was excluded from the feature selection process because of its poor predictive performance.

The four most predictive features, based on their average rank, were the CAP score, CAG repeat length, and 2 rhythm-related speech features: numbers pronounced per second and the SD of the duration of pronounced numbers. Table 2 shows the top 15 most predictive features from the backward speech task for predicting clinical scores.

**Table 2.** Coefficient ranks of demographic and speech features from the backward task for each clinical score. The average rank is calculated as the mean rank across all clinical scores except TFC^a^ (low predictive performance based on explained variance [*R*²]). Features are ordered based on their average rank.

Feature	cUHDRS^b^	TMS^c^	Stroop Word	SDMT^d^	Average rank	TFC^a^
CAP^e^ score	1	1	1	1	1.00	1
Pronounced numbers per second	2	5	3	2	3.00	8
CAG^f^ repetition	3	2	5	7	4.25	2
SD of the duration of pronounced numbers	4	11	2	3	5.00	32
Total number of pronunciation errors	7	4	6	8	6.25	17
Temporal rate of the pronounced numbers	6	3	14	4	6.75	10
Range of the fundamental frequency	5	6	10	13	8.50	4
Temporal rate of silences	9	7	17	5	9.50	6
Pronunciation errors per second	11	8	9	11	9.75	28
Task duration	8	9	7	18	10.50	3
SD of normalized intensity of vocalizations	13	13	8	12	11.50	21
Total number of silences	12	10	20	6	12.00	14
Mean duration of pronounced numbers	10	21	4	16	12.75	13
Ratio of pronunciation errors	14	12	16	9	12.75	12
Temporal rate of the filled pauses	15	16	11	14	14.00	20
Normalized range of intensity of vocalizations	17	17	12	15	15.25	31
Participant age	16	20	15	10	15.25	23

a TFC: Total Functional Capacity.

b cUHDRS: composite Unified Huntington Disease Rating Scale.

c TMS: Total Motor Score.

d SDMT: Symbol Digit Modalities Test.

e CAP: CAG-age-product.

f CAG: cytosine-adenine-guanine.

#### Identifying the Minimal Predictive Features Set

Table 3 shows that using the top 4 ranked features identified in step 1.1 (Table 2) resulted in MAE values that were equal to or lower than those of the initial 63-feature model (column 6 vs column 1). Using only the 2 top-ranked features (CAP score and numbers pronounced per second) was already sufficient for cUHDRS and SDMT to achieve lower MAE compared with the 63-feature model (column 1 vs column 4). Adding CAG repeat length further reduced the MAE for TMS (column 5 vs column 4), whereas incorporating the SD of the duration of pronounced numbers leads to an additional decrease in MAE for SW prediction (column 6 vs column 5).

**Table 3.** Model performance in terms of average MAE^a^ (SD) across 50 cross-validation folds for different sets of features. The goal was to identify the minimal set of predictive features that achieved performance equal to or better than the initial model from our previous study [5], which included 60 speech features, CAP^b^ score, CAG^c^ repeat length, and age (column 1). In columns 3-6, features were added iteratively based on their importance ranking using a standard linear regression model. The top 4 ranked features were, in order, CAP score, backward numbers per second, CAG repeat length, and the SD of backward numbers per second. Column 2 reports performance using only CAP score and CAG repeat length, that is, the 2 demographic features among the top 4 ranked predictors, for comparison. Equivalent performance with the initial 63-feature model was achieved using these 4 features. The last column reports performance using features automatically extracted with Whisper.

Clinical score	Features extracted from annotations used in the regression model						Features extracted using Whisper
	63-feature initial model [5]	Demographic baseline (CAP^b^ score + CAG^c^ repeat length)	Top 1 highest-ranked features	Top 2 highest-ranked features	Top 3 highest-ranked features	Top 4 highest-ranked features (minimal set)	Top 4 highest-ranked features (model automated)
cUHDRS^d^, mean (SD)	2.6 (0.4)	2.7 (0.5)	2.7 (0.5)	2.2 (0.4)	2.2 (0.4)	2.2 (0.3)	2.1 (0.3)
UHDRS-TMS^e^, mean (SD)	10.6 (1.1)	12.0 (1.6)	12.3 (1.8)	10.7 (1.5)	10.4 (1.3)	10.6 (1.3)	10.1 (1.1)
UHDRS-TFC^f^, mean (SD)	1.7 (0.2)	1.5 (0.2)	1.5 (0.2)	1.5 (0.2)	1.4 (0.2)	1.4 (0.2)	1.3 (0.2)
SW^g^, mean (SD)	11.9 (1.9)	14.4 (2.3)	14.5 (2.3)	12.1 (2.1)	12.2 (2.0)	11.6 (1.8)	11.6 (1.7)
SDMT^h^, mean (SD)	8.8 (1.5)	9.7 (1.8)	9.7 (1.7)	8.3 (1.3)	8.3 (1.3)	8.4 (1.3)	8.4 (1.3)

a MAE: Mean Absolute Error

b CAP: CAG-age-product.

c CAG: cytosine-adenine-guanine.

d cUHDRS: composite Unified Huntington Disease Rating Scale.

e UHDRS-TMS: Unified Huntington Disease Rating Scale Total Motor Score.

f UHDRS-TFC: Unified Huntington Disease Rating Scale Total Functional Capacity.

g SW: Stroop Word.

h SDMT: Symbol Digit Modalities Test.

The final 4-feature model outperformed a demographic baseline model based on CAP score and CAG repeat length (column 6 vs column 2; paired *t* test with Bonferroni correction, *P*<.001 for all scores). *R*² values, reported in Table S4 in Multimedia Appendix 1, complement the MAE analysis: for example, the final model achieved a mean *R*² of 0.59 for cUHDRS (vs 0.43 for the baseline) and 0.57 for SW (vs 0.37).

The demographic baseline includes only the demographic features present in the final model to evaluate the incremental value of speech features. Age was not included as a sensitivity analysis showed that adding age to the demographic features did not improve MAE or R² compared to using only CAP score and CAG repeat length (Tables S5 and S6 in Multimedia Appendix 1) and introduced severe multicollinearity (variance inflation factor [VIF] >12 for all features). By contrast, VIF values were acceptable when using only CAP score and CAG repeat length for both the demographic baseline model (VIF=1.2) and the final 4-feature model (maximum VIF of 1.56). This demographic baseline model was used for subsequent comparisons with xHD-Vox to quantify the specific contribution of speech features.

#### Automating the Selected Features’ Annotation Process Using Whisper

Whisper-extracted features and annotations-based features showed Pearson and Spearman correlations of 0.84/0.81 for numbers pronounced per second and 0.42/0.61 for the SD of the duration of pronounced numbers.

The automated pipeline achieved equal or lower MAE values than the speech pathologist-annotated pipeline (column 7 vs column 6 in Table 3), with statistically significant differences observed for TMS and TFC (paired *t* test; Bonferroni corrected; *P*<.001).

The final performance of the automated model yielded relative errors ranging from 12% for SDMT to 40% for TMS, with 13% for TFC, 19% for cUHDRS, and 35% for SW. Relative error was calculated as the MAE (column 7 in Table 3) divided by the mean cohort score (last column in Table 1).

### Model Evaluation Using Longitudinal Data

#### Overview

First, we compared the cross-validation performance of our model obtained in (Table 3, last column) with results previously reported in the literature using comparable evaluation frameworks (Table 4), which is further discussed in the Discussion section.

**Table 4.** Literature review of prediction models and group analyses based on speech markers in Huntington disease. For studies of speech markers of disease, *P* values are indicated when P<.05 for controls vs preHD (Saft) and preHD vs HD (Vogel).

Objective of study	Prediction model				Speech markers of disease		
Authors	This study	Riad et al [5]	Riad et al [13]	Nunes et al [4]	Vogel et al [14]	Skodda et al [15]	Saft et al [16]
Language	French	French	French	English	English	German	German
Type of study	Longitudinal	Cross-sectional	Cross-sectional	Cross-sectional analysis	Cross-sectional	Cross-sectional	Longitudinal (Y0+21 months)
No of participants	181: 41 HD-ISS^a^ 0-1, 33 HD-ISS 2, and 107 HD-ISS 3	103: 87 HDs^b^ and 16 PreHDs^c^	85: 45 HDs, 16 preHDs, and 24 controls	36: 18 HDs, 7 PreHDs, and 11 controls	45: 17 HDs, 13 PreHDs, and 15 controls	56: 28 preHDs and 28 controls	13 preHDs
Number of visits	341	126	85	NR:^d^ follow-up every 3‐6 months for 3 years	45	56	26
Automated?	Yes	No (speech pathologist annotations)	Yes (acoustic features)	Automated using BioDigit Speech (proprietary ASR^e^)	Yes (purely acoustic)	Yes (purely acoustic)	Yes (purely acoustic)
Tasks	Counting backward	Counting backward + forward	Maximum phonation	Passage reading, counting forward (1-20), counting backward (50-30 by increments of 3), and used for classification only.	Passage reading, monologue with positive content, and automated sample (days of the week)	Reading, sustained phonation, maximum syllable repetition capacity, and steadiness of syllable repetition.	Reading, sustained phonation, maximum syllable repetition capacity, and steadiness of syllable repetition.
Task duration	≤40 seconds	≤80 seconds	3 seconds	≤2 minutes	NR (presumably >2 minutes)	NR (presumably >2 minutes)	NR (presumably >2 minutes)
Number of features	4	63	16	14	6	6	6
Features	CAP^f^ score Numbers pronounced per second CAG^g^ repeat length SD of numbers pronounced per second	Sixty speech features (listed in Table S1 in Multimedia Appendix 1). CAP score CAG repeat length Age	Phonatory features and modulation power spectrum features	Features for reading and counting task: rhythm features, sequence-error perseverations, and articulatory and phonatory deficiencies. Additional features for counting task: correct and incorrect counts and their ratio.	Total speech time (*P*<.01) Total silence time (*P*<.01) % silence Speech rate (*P*<.0001) F0 coefficient of variation Alpha ratio	Net speech rate,(*P*<.0001) Pause ratio Vowel keeping time (VKT) and MaxSylRep COV1^h^ (*P*<.0001) COV2^i^ IntDur: interval duration (ms) PatiRatio^r^	Net speech rate Pause ratio VKT and MaxSylRep COV1 ^h^ COV2 ^i^ IntDur: interval duration (ms; *P*<.001) PatiRatio^r^ (*P*<.001)
Model	Linear regression	Elastic net	Elastic net	Random forest	—^j^	—	—
MAE^k^, mean (SD)							
cUHDRS^l^	2.1 (0.3)	2.4 (0.4)	2.78 (0.5)	NA^m^	—^j^	—	—
TMS^n^	10.1 (1.1)	12 (1.8)	13.14 (1.7)	9.64	—	—	—
TFC^o^	1.3 (0.2)	1.3 (0.2)	1.64 (0.3)	2.43	—	—	—
SW^p^	11.6 (1.7)	13.3 (2.2)	NA	NA	—	—	—
SDMT^q^	8.4 (1.3)	8.9 (1.8)	NA	NA	—	—	—

a HD-ISS: Huntington Disease Integrated Staging System.

b HD: Huntington Disease.

c PreHD: premanifest stage.

d NR: not reported.

e ASR: automatic speech recognition.

f CAP: CAG-age-product.

g CAG: cytosine-adenine-guanine.

h COV1: relative coefficient of variation for single syllable repetition.

i COV2: relative coefficient of variation of the pairs of syllables.

j Not applicable.

k MAE: mean absolute error.

l cUHDRS: composite Unified Huntington Disease Rating Scale.

m Not available.

n TMS: Total Motor Score.

o TFC: Total Functional Capacity.

p SW: Stroop Word.

q SDMT: Symbol Digit Modalities Test.

r PatiRatio: average interval duration of syllables (pa) divided by average interval duration of syllable (ti)

#### Model Equations

The regression coefficients of the xHD-Vox model, calibrated on the training set, are provided in Table 5.

**Table 5.** Regression coefficients of the xHD-Vox model: a standard linear regression model predicting clinical scores. All speech features were extracted from the backward counting task. Variables were not normalized before regression for reproducibility purposes. Exact *P* values are reported when *P*≥.001. As an example, the regression equation for predicting cUHDRS^a^ is: cUHDRS=33.05−0.12 × CAP^b^ score + 2.5 × numbers pronounced per second − 0.27 × CAG^c^ repetition − 2.32 × SD of numbers pronounced per second. Definition of speech features: task duration = time between the first and last spoken numbers (from 20 to 1). Numbers pronounced per second = total number of numbers spoken divided by task duration (including perseverations). SD of numbers pronounced per second = variability in duration across individual spoken numbers.

Feature	cUHDRS^a^	TMS^d^	TFC^e^	Stroop Word	SDMT^f^
Intercept	33.05^g^ (*P*≤.001)	−78.10^g^ (*P*≤.001)	19.52^g^ (*P*≤.001)	164.87^g^ (*P*≤.001)	97.33^g^ (*P*≤.001)
CAP^b^ score	−0.12^g^ (*P*≤.001)	0.45^g^ (*P*≤.001)	−0.04^g^ (*P*≤.001)	−0.56^g^ (*P*≤.001)	−0.42^g^ (*P*≤.001)
Numbers pronounced per second	2.50^g^ (*P*≤.001)	−8.40^g^ (*P*≤.001)	0.94^g^ (*P*≤.001)	14.3^g^ (*P*≤.001)	8.25^g^ (*P*≤.001)
CAG^c^ repetition	−0.27^g^ (*P*≤.001)	1.44^g^ (*P*≤.001)	−0.11^h^ (*P*=.009)	−1.15^g^ (*P*≤.001)	−0.66^i^ (*P*=.02)
SD of numbers pronounced per second	−2.32^g^ (*P*≤.001)	10.2^g^ (*P*≤.001)	−1.27^h^ (*P*=.002)	−10.54^h^ (*P*=.001)	−5.06^i^ (*P*=.046)

a cUHDRS: composite Unified Huntington Disease Rating Scale.

b CAP: CAG-age-product.

c CAG: cytosine-adenine-guanine.

d TMS: Total Motor Score.

e TFC: Total Functional Capacity.

f SDMT: Symbol Digit Modalities Test.

g *P*≤.001.

h *P*≤.01.

i *P*<.05.

#### Model Performance on the Test Set

**Figure 2.**
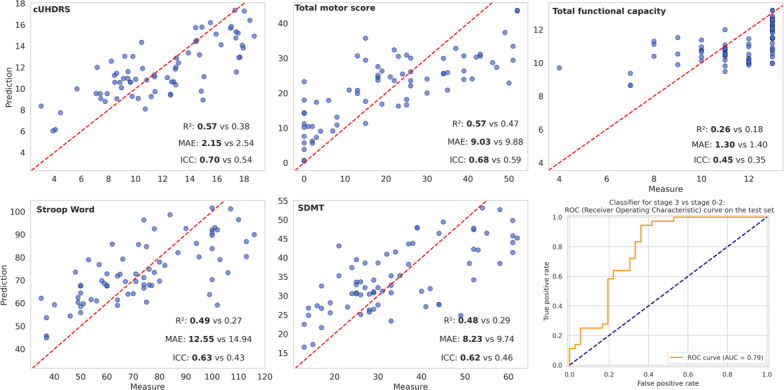
Model performance on the testing set (72 visits, 24 participants). Predicted clinical scores vs measured values (blue dots). The red line represents perfect predictions. The mean absolute error (MAE) corresponds to the vertical distance between a point and this line. The explained variance (*R*²), MAE, and intraclass correlation coefficient (ICC) of our model (bold) vs those obtained with a demographic baseline (cytosine-adenine-guanine (CAG)-age-product [CAP] score + CAG repetition). The bottom-right panel displays the Receiver Operating Characteristic curve for a logistic classifier distinguishing Huntington Disease Integrated Staging System stage 3 from stages 0‐2, based on CAP score and speech rate. Refer to Figure S3 in Multimedia Appendix 1 for comparison of the classifier’s performance compared to CAP score alone. cUHDRS: composite Unified Huntington Disease Rating Scale; HD-ISS: Huntington Disease Integrated Staging System; SDMT: Symbol Digit Modalities Test.

The *R*² values of xHD-Vox on the test set were 0.57 for cUHDRS and TMS, 0.49 for SW, and 0.48 for SDMT (Figure 2). The lowest *R*² was observed for TFC (0.26).

Compared with a demographic baseline model including CAP score and CAG repeat length, xHD-Vox improved cUHDRS prediction, increasing *R*² by 50% and reducing MAE by 15%. The largest gains were observed for cognitive outcomes, with relative *R*² increases of 81% for SW and 66% for SDMT, along with corresponding MAE reductions of 16% and 13%, respectively.

Based on the model’s cross-validation results in Table 3 (last column), the test set MAE for cUHDRS matched expectations (2.1), equal to the average MAE across the 50 folds. For Symbol Digit Modality and TMS, the test-set MAEs were lower than expected (8.2 vs 8.4 and 9.0 vs 10.1, respectively). In contrast, SW showed poorer test-set performance, with a higher MAE than during cross-validation (12.5 vs 11.6).

Mean and median absolute errors per visit remained stable over the 2-year follow-up (Figure S4 in Multimedia Appendix 1). MAE varied by HD-ISS stage (Figure S5 in Multimedia Appendix 1). For example, for cUHDRS, the MAE decreased from 2.6 at stage 0‐1 to 1.7 at stage 2 before increasing to 2.2 at stage 3.

#### Functional Classification

Model performance for TFC prediction was modest (*R*²=0.26), likely reflecting the limited variance in TFC scores (mean 11.1, SD 2.4). However, speech rate was significantly lower in participants at HD-ISS stage 3 compared to earlier stages (Figure S6 in Multimedia Appendix 1; 1-way ANOVA followed by post hoc Tukey test; *P*<.001).

We therefore investigated whether speech rate could serve as a marker of functional impairment and trained a logistic regression classifier using CAP score and speech rate as the 2 predictors (methods and Figure S3 in Multimedia Appendix 1). The model distinguished stage 3 from stages 0‐2 with good performance on the held-out test set: recall=0.83, accuracy=0.73, and ROC AUC=0.79 (bottom right panel in Figure 2). For comparison, a model based only on CAP score achieved recall=0.68, accuracy=0.68, and ROC AUC=0.74.

#### Longitudinal Change

We conducted repeated-measures 2-way ANOVAs with time (Y0, Y1, and Y2) and type of measure (clinical vs xHD-Vox) as within-subject factors for all scores (Table 6). A significant main effect of time was observed for all scores (*P*<.001 for all scores), with no significant time × type interaction, indicating similar temporal trajectories for clinical measures and xHD-Vox predictions. Post hoc Tukey tests revealed significant differences between Y0 and Y2 for all scores, and between all time points (Y0, Y1, and Y2) for cUHDRS and SW. Mean scores decreased consistently across time points for both clinical and xHD-Vox measures, demonstrating a statistically significant longitudinal decline captured by both measures. No significant main effect of type of measure was observed. Detailed ANOVA results for the main effect of type and the time × type interaction are provided in Table S7 in the Multimedia Appendix 1. These results demonstrate that xHD-Vox reliably captures longitudinal clinical decline over a 2-year follow-up.

**Table 6.** Mean clinical and xHD-Vox scores over time and results of repeated-measures 2-way ANOVA for the main effect of time. Repeated-measures 2-way ANOVA was conducted for each clinical score, with factors time (Y0, Y1, and Y2) and type of measure (clinical vs xHD-Vox). All ANOVAs were corrected for multiple comparisons using the Bonferroni method. Results for the main effect of time are reported here. Post hoc Tukey tests were performed for pairwise comparisons when the time effect was significant.

Clinical score	Clinical measures			xHD-Vox measure			Time effect (repeated-measured ANOVA)			
	Y0, mean (SD)	Y1, mean (SD)	Y2, mean (SD)	Y0, mean (SD)	Y1, mean (SD)	Y2, mean (SD)	*F*(1, 2)	*P* value	np²^a^	Post hoc (Tukey, Bonferroni corrected)
cUHDRS^b^	12.75 (3.65)	11.99 (4.02)	11.29 (4.41)	12.17 (2.49)	11.73 (2.83)	11.51 (2.57)	17.83 (2, 46)	<.001	0.02	Y1 vs Y2, Y1 vs Y0, and 2 vs Y0
UHDRS-TMS^c^	19.25 (15.11)	21.29 (16.72)	23.33 (18.10)	20.06 (9.23)	21.74 (10.50)	22.61 (9.58)	8.76 (2, 46)	.001	0.01	Y1 vs Y2 and Y2 vs Y0
UHDRS-TFC^d^	11.88 (1.48)	11.38 (1.88)	11.04 (2.30)	11.15 (0.97)	10.97 (1.12)	10.88 (1.02)	8.85 (2, 46)	.001	0.02	Y1 vs Y0 and Y2 vs Y0
SW^e^	77.83 (22.00)	73.75 (21.30)	69.04 (22.10)	75.43 (12.56)	73.26 (14.40)	72.21 (13.05)	14.23 (2, 46)	<.001	0.02	Y1 vs Y2, Y1 vs Y0, and Y2 vs Y0
SDMT^f^	36.21 (13.35)	34.42 (14.04)	32.71 (14.80)	35.94 (8.30)	34.54 (9.30)	33.76 (8.50)	9.94 (2, 46)	<.001	0.01	Y2 vs Y0

a ηp²: partial eta squared.

b cUHDRS: composite Unified Huntington Disease Rating Scale.

c UHDRS-TMS: Unified HuntingtonRating Scale Disease Rating Scale Disease Rating Scale Total Motor Score.

d UHDRS-TFC: Unified HuntingtonRating Scale Disease Rating Scale Total Functional Capacity.

e SW: Stroop Word.

f SDMT: Symbol Digit Modalities Test.

The magnitude of the 1-year and 2-year decline at the group level is reported in Table 7, which presents mean changes and their 95% CIs. Corresponding plots are shown in Figure S7 in Multimedia Appendix 1 for illustrative purposes. For all clinical scores, the mean 2-year decline measured by clinicians was approximately twice the 1-year decline, indicating a consistent and approximately linear progression over time. Predicted changes derived from xHD-Vox fell within the 95% CIs of the corresponding clinician-assessed changes for all scores except the 2-year change in SW. These results suggest that xHD-Vox provides a group-level estimate of the magnitude of longitudinal clinical decline.

**Table 7.** One-year and 2-year mean changes in clinical scores for model predictions and clinician assessments on the test set (N=24). Predicted mean changes are compared with the clinician-derived 95% CIs to evaluate alignment between model outputs and expert ratings. For example, the predicted 1-year change in cUHDRS^a^ (−0.44) falls within the 95% CI of the clinician assessments (−1.3 to −0.2).

Clinical score	1-year change, mean (95% CI)		2-year change, mean (95% CI)	
Clinician	Predicted	Clinician	Predicted
cUHDRS^a^	−0.8 (−1.3 to −0.2)	−0.44 (−0.9 to −0.0)	−1.5 (−2.3 to −0.6)	−0.7 (−1.1 to −0.3)
TMS^b^	2.0 (−1.2 to 5.3)	1.7 (0.2 to 3.2)	4 (0.1 to 8.0)	2.6 (1.1 to 4.0)
TFC^c^	−0.5 (−0.9 to −0.1)	−0.2 (−0.4 to 0)	−0.8 (−1.5 to −0.1)	−0.3 (−0.4 to −0.1)
SW^d^	−4.1 (−7.4 to −0.8)	−2.2 (−4.4 to 0.1)	−8.8 (−13.6 to −3.9)	−3.2 (−5.4 to −1.1)
SDMT^e^	−1.8 (−3.8 to 0.2)	−1.4 (−2.6 to −0.2)	−3.5 (−6.2 to −0.8)	−2.2 (−3.4 to −1)

a cUHDRS: composite Unified Huntington Disease Rating Scale.

b TMS: Total Motor Score.

c TFC: Total Functional Capacity.

d SW: Stroop Word.

e SDMT: Symbol Digit Modalities Test.

**Figure 3.**
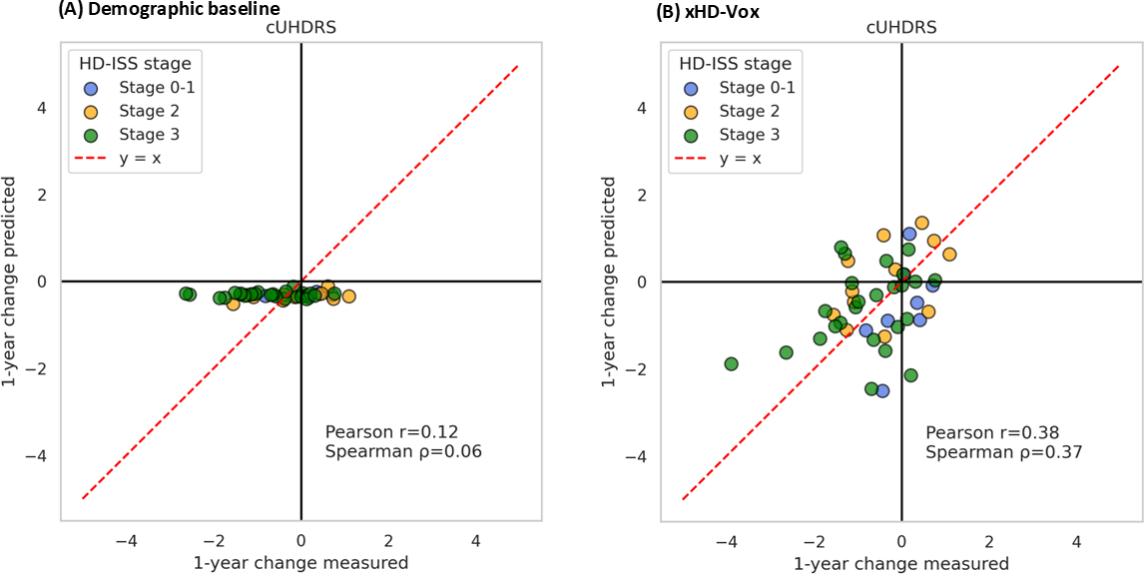
One-year change in composite Unified Huntington Disease Rating Scale (cUHDRS) predicted vs clinically measured for xHD-Vox (panel B) and the demographic baseline model (panel A). The red line represents the line of perfect agreement (y = x). Pearson and Spearman correlation coefficients are reported. The main analysis includes 45 observations, with 3 influential outliers removed; a sensitivity analysis including all 48 observations is shown in Figure S8 in Multimedia Appendix 1. HD-ISS: Huntington Disease Integrated Staging System.

Figure 3 compares predicted 1-year changes in cUHDRS with clinically measured changes at the individual level for both xHD-Vox and the demographic baseline model (45 observations; 3 influential outliers removed). A sensitivity analysis including all 48 observations is reported in Figure S8 in Multimedia Appendix 1 and discussed in the following paragraphs. The demographic baseline captured little intraindividual variability, with Pearson and Spearman correlations of 0.12 and 0.06, respectively, and produced identical slopes across participants.

In contrast, xHD-Vox exhibited greater interindividual variability, with Pearson and Spearman correlations of 0.38 and 0.37, respectively. The model also captured positive changes in some participants at earlier disease stages (HD-ISS 0‐2), as illustrated in Figure 3 and in the individual trajectories shown in Figure S9 in Multimedia Appendix 1.

A sensitivity analysis including all 48 observations (24 participants with 2 annual change measures each) showed that correlation estimates were strongly influenced by 3 influential outliers. When all data points were included, the Pearson correlation was close to 0, whereas the Spearman correlation remained higher (ρ=0.17). The discrepancy between Pearson and Spearman correlations suggested a monotonic relationship that was disproportionately affected by a small number of extreme values, as identified in Figure S8 in Multimedia Appendix 1.

## Discussion

This study presented an interpretable, automated speech-based model, xHD-Vox, for monitoring clinical progression in HD. Building on Riad et al [5] prior work using counting tasks, we reduced the feature set from 63 to 4 key predictors while maintaining comparable MAE: CAP score, CAG repeat length, and rhythm-related measures—speech rate (numbers pronounced per second) and its SD. These 2 rhythm features were automatically derived from Whisper’s transcriptions and timestamps, an open-source ASR model [8].

xHD-Vox was trained on data from 157 participants and evaluated on a longitudinal cohort of 24 participants monitored annually during 2 years. On this test set, xHD-Vox explained 57% of the variance in cUHDRS, representing a 50% improvement over the demographic baseline. The MAE was 2.2, with an ICC of 0.70—lower than the 0.92 reported for clinicians [17], but promising for a fully automated tool. Similar results were obtained for TMS (MAE=9.0; ICC=0.68 vs ICC=0.84 for clinicians [18]).

Cognitive outcomes also showed significant improvement relative to the demographic baseline, with an R² increase of 66% for SDMT, reaching 0.48. To assess clinical relevance, we compared our MAE values with normative benchmarks from Mills et al [19]. The model’s MAE for SW (12.5) approximated the difference between the 50th and 5th percentiles (11) for highly educated 50-year-olds, a demographic representative of our cohort. Similarly, the MAE for SDMT (8.2) closely matched the normative spread (8).

Although the linear regression model yielded a relatively low *R*² for TFC (0.26), a logistic regression using speech rate and CAP score effectively classified HD-ISS stage 0‐2 vs stage 3 with an ROC AUC of 0.79, supporting its potential utility in detecting functional decline.

Longitudinal analyses using repeated-measures ANOVAs with post hoc Tukey tests confirmed a significant decline over the 2-year follow-up in the test set for both clinician-assessed measures and xHD-Vox predictions. At the group level, the mean 1-year and 2-year changes predicted by xHD-Vox were consistent with clinically measured changes, falling within the corresponding 95% CIs. Exploratory individual-level analyses showed that xHD-Vox reasonably captured longitudinal 1-year change compared with a demographic baseline model, although results were sensitive to a small number of influential outliers. Collectively, these findings support the potential of xHD-Vox to model longitudinal clinical trajectories, while underscoring the need for larger and more diverse test samples to further assess robustness at the individual level.

Compared to prior speech studies in HD (summarized in Table 4), our model stands out for its larger cohort (n=181 gene carriers), improved performance, and longitudinal scope. In terms of performance, the MAE here was reduced for SW and TMS by 13% and 16%, respectively, relative to Riad et al [5]. For TFC, a 46% improvement was achieved compared with Nunes et al [4], although this should be interpreted cautiously, considering the difference in sample size in cross-validation (n=25 in their study). The only published longitudinal study did not model clinical outcomes but focused on the decline of speech biomarkers over time in premanifest participants (n=13) [16], which precludes direct comparison [14,16].

Unlike most previous studies, we relied on a backward counting task, a task widely conceptualized as a working memory task. Individual differences in working memory capacity are known to covary with processing speed and broader cognitive efficiency [20,21], which are core determinants of SDMT and SW performance. In addition, backward counting and the SW task both rely on inhibitory control processes: inhibition of the overlearned forward number sequence in backward counting and inhibition of the automatic reading response in the Stroop task. Thus, rhythm-related speech features likely capture these shared cognitive efficiencies, explaining their predictive value for broader cognitive performance beyond working memory alone. Speech rate was also demonstrated to be a marker of disease progression in HD [14,15]. Consistent with Vogel et al [14], we observed a reduction in speech rate in stage 3 participants compared with controls and earlier-stage patients (Figure S6 in Multimedia Appendix 1). We did not detect significant differences between stages 0‐1 and controls in our measure of speech rate, in agreement with Vogel et al [14] but in contrast to findings reported by Skodda et al [15] (refer to Table 4 for sample sizes in each group and study). Although speech rate in stage 2 participants was lower than in stages 0‐1, this difference was not statistically significant in our cohort (*P*=.07), whereas it was significant in Vogel et al [14]. Differences across studies may reflect variability in sample size, statistical approaches, and, importantly, the experimental task used. Indeed, Vogel et al [14] highlighted the task-dependent nature of speech rate as a biomarker, showing weak correlations between speech rate and disease burden score in a days-of-the-week recitation task (*R*=−0.22), but stronger correlations for reading (*R*=−0.68). These considerations underscore the importance of task selection when using speech rate as a cognitive and disease-related marker.

Our model can be implemented in mobile apps for remote monitoring. We provided all model equations and code on GitHub, ensured clinical interpretability through identified features, and automated feature extraction using the open-source algorithm Whisper. Whisper produced accurate transcriptions and sequence-level timestamps, resulting in high agreement with speech pathologists’ annotations for speech rate. Agreement was lower for the SD of numbers pronounced, which relies on word-level timestamps, but this limitation does not substantially compromise overall model performance. Here, the use of Whisper addressed reproducibility concerns raised by a previous study [4] that relied on proprietary ASR systems or performance issues of acoustic-based automated speech analysis [13]. Despite being trained on general speech, Whisper has demonstrated strong performance for dysarthric speech, including in alpha-synucleopathies (Pearson correlations ranging from 0.6 to 0.94 depending on linguistic features) [22]. Fine-tuning Whisper for dysarthric speech (eg, CrisperWhisper [23]) or leveraging its audio encoder opens opportunities for other predictive approaches [24].

XHD-Vox opens the door for several applications. First, it could be used in underserved areas. About 40 % of individuals with Parkinson disease currently do not receive care from a specialist [25]. Given that HD is a rare disease, we anticipate that the situation may be similar or even worse. Like Parkinson disease, HD specialists are concentrated in urban medical centers, while patients are geographically dispersed, making travel to these centers more difficult due to disability and age. By offering a simple and ecological approach for tracking disease progression, the need for clinic visits may decrease, whereas increasing the number of people benefiting from appropriate monitoring. Second, by allowing for frequent measurements of disease severity, xHD-Vox could assist clinicians in following patients, especially during crises such as COVID-19, when patients could not attend their annual visits. Third, our approach could be used for screening patients’ disease severity for clinical trials, especially between stages 0‐2 and stage 3, using our classifier.

However, xHD-Vox has not yet been tested in other languages. Fahed et al [26] conducted the only multilingual study (English, Spanish, and Polish) on acoustic biomarkers in HD, focusing on reading tasks, syllable repetition, and sustained vowels. They showed that all temporal features are language-dependent in reading tasks. In Parkinson disease, the articulation rate also differed across languages [27]. With Whisper supporting 96 languages, adapting xHD-Vox to a new language would require fewer than 100 participants to achieve 95% accuracy (Figure S2 in Multimedia Appendix 1). While we have tested our model on an unseen sample (test set) with good results, replication in cohorts from other centers would strengthen generalizability. Additionally, testing real-world data collection by recording patients on their mobile phones, as done for Parkinson disease [28], would validate the approach for remote monitoring. Building on this framework, we have developed an Android prototype of xHD-Vox, currently under internal testing, to explore its potential for mobile deployment and remote monitoring.

In conclusion, we provide novel insights into the robustness of an automated speech-based clinical estimation model for longitudinal monitoring of HD gene carriers, xHD-Vox. Its strengths lie in its automation and interpretability, facilitating deployment on mobile platforms and acceptance by clinicians. This work represents a step forward in telemedicine for HD monitoring, with the potential to detect disease progression, particularly in regions with limited medical access or during periods of health care disruption, such as lockdowns.

## Supplementary material

10.2196/83838 Multimedia Appendix 1 Additional figures, tables, and methods.

10.2196/83838 Checklist 1 TRIPOD+AI checklist.

## Abbreviations

ASR: automatic speech recognition
CAG: cytosine-adenine-guanine
CAP: CAG-age-product
HD: Huntington disease
HD-ISS: Huntington Disease Integrated Staging System
ICC: intraclass correlation coefficient
MAE: mean absolute error
PRISMA: Preferred Reporting Items for Systematic Reviews and Meta-Analyses
ROC AUC: Receiver Operating Characteristic–Area Under the Curve
SDMT: Symbol Digit Modalities Test
SW: Stroop Word
TFC: Total Functional Capacity
TMS: Total Motor Score
TRIPOD+AI: Transparent Report of a Multivariable Prediction Model for Individual Prognosis or Diagnosis
UHDRS: Unified Huntington Disease Rating Scale
VIF: variance inflation factor
