# Supplementary material for: xHD-Vox, an Automated Speech Model for Estimating Motor and Cognitive Scores in Huntington Disease: Development and Longitudinal Validation
Source: JMIR Neurotechnol. 2026 Jul 8;5:e83838. doi: 10.2196/83838 (PMC13345346; doi:10.2196/83838)
Supplement: Multimedia Appendix 1 [file neuro-v5-e83838-s001.docx]

**Logistic regression to classify HD-ISS Stage 3 vs HD-ISS Stages 0,1, and 2**

Although regression models showed limited performance in predicting TFC, we investigated whether our features could still detect functional impairment. Consistent with previous findings [15], participants at HD-ISS Stage 3 exhibited significantly lower speech rates compared to earlier stages and controls (Figure S6; one-way ANOVA with post hoc Tukey test, *P < .001*).

We trained a logistic regression model to classify Stage 3 versus Stages 0-2 using two predictors: CAP score and speech rate (measured as numbers pronounced per second). This feature selection was supported by cross-validation results shown in Figure S3. The model was trained using 50-fold cross-validation on the training set (157 participants; 80% training, 20% validation per fold). Performance was evaluated on the held-out test set using recall, accuracy, and ROC AUC. Accuracy reflects the proportion of correct classifications made by the logistic regression model. Recall, in this context, measures the proportion of true Stage 3 cases correctly identified. The ROC AUC indicates the model’s ability to discriminate between Stage 3 and Stages 0-2, regardless of the classification threshold.

**Tables and Figures**

| Dimension | Speech/language feature |
| --- | --- |
| Articulatory and phonatory deficiencies | Total number of pronunciations errors |
|  | Ratio of pronunciation errors |
|  | Pronunciation error per second |
|  | Mean intelligibility based on non-intrusive normed speech-to-reverberation modulation energy ratio metric |
|  | SD of the fundamental frequency |
|  | Range of the fundamental frequency |
|  | SD of normalized intensity of vocalizations |
|  | Normalized range of intensity of vocalizations |
| Rhythm and temporal statistics | Task duration |
|  | Temporal rate of the pronounced numbers |
|  | Mean duration of pronounced numbers |
|  | Pronounced numbers per second |
|  | SD of the duration of pronounced numbers |
|  | Phones per second |
|  | TR of the silences |
|  | Mean duration of silences |
|  | SD of the duration of silences |
|  | Total number of silences |
| Sequence errors and perseverations | Levenshtein distance between the pronounced numbers and the target sequence (1,2,....,19,20) |
|  | Gestalt similarity between the pronounced numbers and the target sequence (1,2,....,19,20) |
|  | Levenshtein distance between the pronounced phones and the target sequence (phones of 1, phones of 2, …, phones of 19, phones of 20) |
|  | Gestalt similarity between the pronounced phones and the target sequence (phones of 1, phones of 2, …, phones of 19, phones of 20) |
|  | Total number of pronounced numbers |
|  | Total number of pronounced phones |
| Collateral track additions | Total number of involuntary/abnormal vocalizations |
|  | Involuntary/Abnormal vocalizations per second |
|  | Temporal rate of the involuntary/abnormal vocalizations |
|  | Total number of filled pauses |
|  | Filled pauses per second |
|  | Temporal rate of the filled pauses |

**Table S1**:List of speech and language features extracted from the recitation of numbers- SD stands for standard deviation, Temporal rate is defined as the ratio of the total time of a specific class on the total time to perform the task.


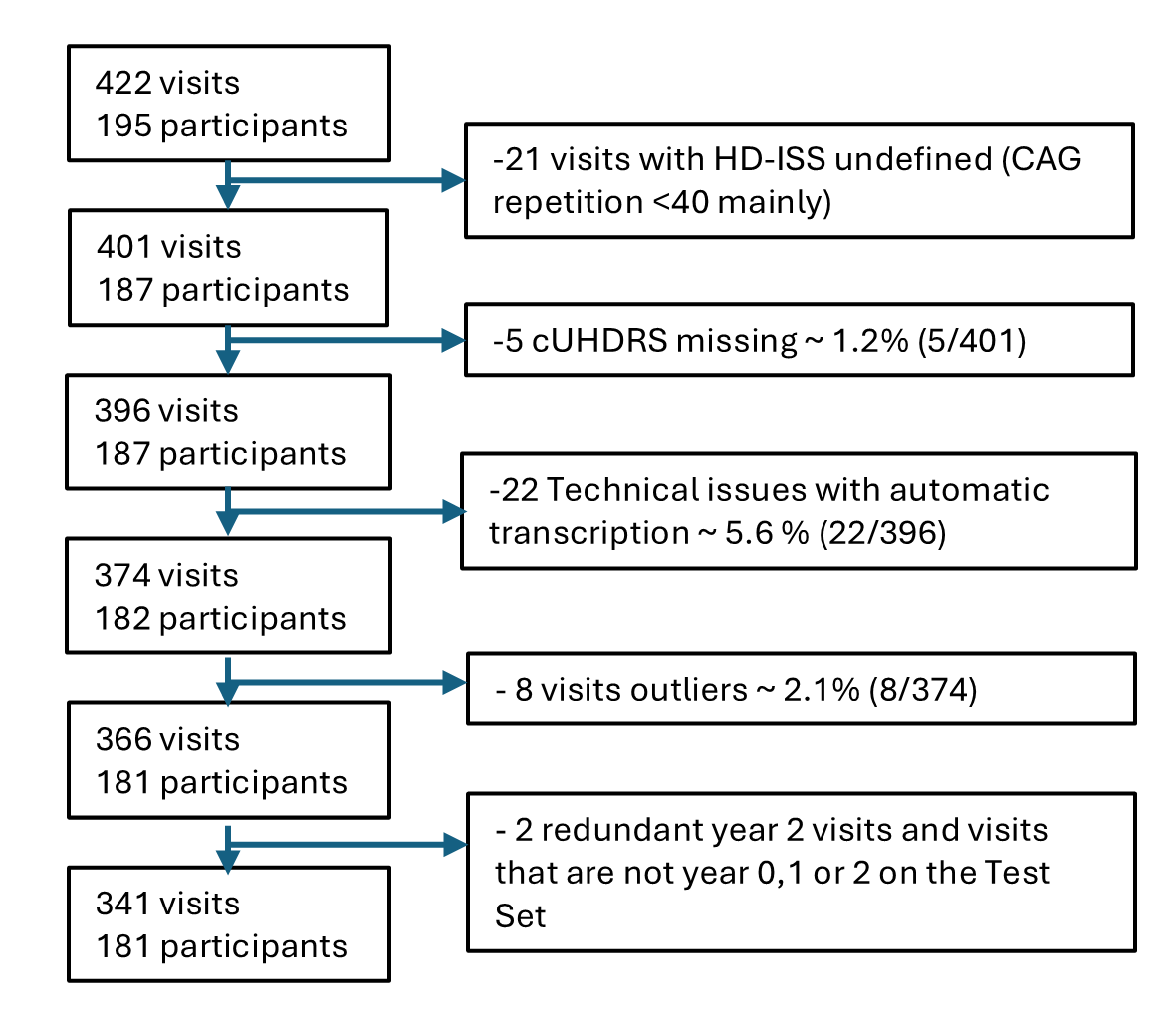


**Figure S1**. PRISMA Flow Diagram of Study Inclusion - This diagram summarizes the inclusion process for 422 visits with audio recordings, drawn from three clinical research cohorts: BIO-HD, REPAIR-HD, and MIG-HD.

A known limitation of these cohorts is a selection bias toward participants more likely to complete lengthy assessments. Outliers were defined as visits where the variable “numbers pronounced per second” fell outside the interquartile range (25%-75%) within each HD-ISS stage. This criterion helped identify potential anomalies in the Whisper transcription output.

Only 15 visits (4%) came from the MIG-HD cohort. Due to poor audio quality-mainly from video recordings dating back to the early 2000s-only 30% of MIG-HD recordings were usable with the Whisper model.

| 63-features  Initial model^5^ | Backward speech features | Forward speech features |  |
| --- | --- | --- | --- |
| cUHDRS | 2.6 (0.4) | 2.6 (0.4) | 2.9 (0.5) |
| UDHRS TMS | 10.6 (1.1) | 10.6 (1.2) | 11.6 (1.4) |
| UHDRS TFC | 1.7 (0.2) | 1.7 (0.2) | 1.7 (0.2) |
| STROOP WORD | 11.9 (1.9) | 12.1 (1.8) | 13.8 (2.2) |
| SDMT | 8.8 (1.5) | 8.9 (1.4) | 9.7 (1.7) |

**Table S2**. Model performance (average MAE ± SD over 50 validation folds) using different feature sets: all 63 features (from both tasks), 30 backward task features+ 3 demographic, and 30 forward task features+ 3 demographics. Performance with backward-only features was equivalent to that with all features.

| 63-features  Initial model^5^ | Backward speech features | Forward speech features |  |
| --- | --- | --- | --- |
| cUHDRS | 0.48 (0.09) | 0.48 (0.09) | 0.40 (0.10) |
| UDHRS TMS | 0.54 (0.11) | 0.53 (0.10) | 0.48 (0.11) |
| UHDRS TFC | 0.13 (0.10) | 0.13 (0.10) | 0.12 (0.09) |
| STROOP WORD | 0.55 (0.11) | 0.55 (0.10) | 0.41 (0.14) |
| SDMT | 0.41 (0.16) | 0.41 (0.15) | 0.31 (0.16) |

**Table S3**. Model performance (average R² over 50 validation folds) using different feature sets: all 63 features (from both tasks), 30 backward task features+ 3 demographic, and 30 forward task features+ 3 demographics. Performance with backward-only features was equivalent to that with all features.

|  | | Features extracted from annotations used in the regression model | | | | | Features extracted using Whisper |  |
| --- | --- | --- | --- | --- | --- | --- | --- | --- |
| 63-features  Initial model [5] | Demographic : CAP score + CAG repetition length | | Top 1 ranked features | Top 2 ranked features | Top 3 ranked features | Top 4 ranked features  **Minimal set** | Top 4 ranked features  **Model automated** |  |
| cUHDRS | 0.48 (0.09) | 0.43 (0.15) | | 0.41 (0.15) | 0.57 (0.13) | 0.60 (0.12) | 0.59 (0.12) | 0.63 (0.10) |
| UDHRS TMS | 0.54 (0.11) | 0.42 (0.14) | | 0.40 (0.15) | 0.51 (0.13) | 0.54 (0.12) | 0.53 (0.12) | 0.58 (0.10) |
| UHDRS TFC | 0.13 (0.10) | 0.28 (0.14) | | 0.25 (0.14) | 0.30 (0.13) | 0.33 (0.12) | 0.32 (0.12) | 0.39 (0.13) |
| SW | 0.55 (0.11) | 0.37 (0.16) | | 0.37 (0.15) | 0.52 (0.15) | 0.53 (0.15) | 0.57 (0.13) | 0.55 (0.13) |
| SDMT | 0.41 (0.16) | 0.27 (0.18) | | 0.28 (0.17) | 0.46 (0.17) | 0.47 (0.17) | 0.46 (0.17) | 0.45 (0.17) |

**Table S4**. Model performance in terms of average R2 (SD) across 50 cross-validation folds for different sets of features. The goal is to identify the minimal set of predictive features that achieves performance equal to or better than the initial model from our previous study [5], which included 60 speech features, CAP score, CAG repeat length, and age (Column 1). In columns 2 to 5, features were added iteratively based on their importance ranking in Table 2in a standard linear regression model. The top four ranked features were in their respective order: CAP score, backward numbers per second, CAG repeat length, and the standard deviation of backward numbers per second. Equivalent performance was achieved using these four features. The last column reports performance using features automatically extracted with Whisper.

|  | Model automated | CAP score | **CAP score + CAG repeat length** | **CAP score + Age** | **CAP score + CAG repeat length +Age** | **Age + CAG repeat length** | **age** | **CAG repeat length** |
| --- | --- | --- | --- | --- | --- | --- | --- | --- |
| **cUHDRS** | 2.1 (0.3) | 2.7 (0.5) | 2.7 (0.5) | **2.6 (0.5)** | 2.7 (0.5) | 2.9 (0.5) | 3.9 (0.6) | 3.6 (0.5) |
| **UDHRS TMS** | 10.1 (1.1) | 12.3 (1.8) | 12.0 (1.6) | **11.9 (1.6)** | 12.1 (1.6) | 12.8 (1.8) | 17.6 (2.5) | 15.8 (1.8) |
| **UHDRS TFC** | 1.3 (0.2) | 1.5 (0.2) | 1.5 (0.2) | **1.5 (0.2)** | 1.5 (0.2) | 1.5 (0.2) | 1.9 (0.2) | 1.7 (0.2) |
| **STROOP WORD** | 11.6 (1.7) | 14.5 (2.3) | 14.4 (2.3) | **14.3 (2.3)** | 14.3 (2.4) | 15.3 (2.1) | 19.8 (3.1) | 19.0 (2.6) |
| **SDMT** | 8.4 (1.3) | 9.7 (1.7) | 9.7 (1.8) | **9.7 (1.8)** | 9.9 (1.8) | 10.3 (1.8) | 12.9 (2.2) | 12.4 (2.0) |

**Table S5**. Model performance measured by average mean absolute error (MAE) ± standard deviation (SD) across 50 cross-validation folds for different sets of demographic features. Column 1 shows the performance of our automated model for comparison. Among the demographic feature combinations, CAP score + Age achieved the lowest MAE, showing a statistically significant, but small, improvement over CAP score + CAG repeat length (maximum difference: err0.1) for cUHDRS and Stroop Word (P < .001, paired t-test, Bonferroni corrected). This combination was not used in the main analysis, which relied on CAP score + CAG repeat length, the two features included in xHD-Vox, to assess the incremental value of the automated model relative to demographic features. Despite this, the automated model still significantly outperformed the CAP score + Age model (P < .001, paired t-test, Bonferroni corrected).

|  | Model automated | CAP score | CAP score + CAG repeat length | CAP score + Age | CAP score + CAG repeat length+Age | age + CAG repeat length | age | CAG repeat length |  |
| --- | --- | --- | --- | --- | --- | --- | --- | --- | --- |
|  |  |  |  |  |  |  |  |  |  |
| **cUHDRS** | 0.59 (0.12) | 0.41 (0.15) | **0.43 (0.15)** | **0.43 (0.16)** | 0.42 (0.16) | 0.37 (0.13) | -0.09 (0.14) | 0.07 (0.16) |  |
| **UDHRS TMS** | 0.53 (0.12) | 0.40 (0.15) | **0.42 (0.14)** | **0.42 (0.15)** | 0.41 (0.15) | 0.38 (0.12) | -0.08 (0.12) | 0.10 (0.15) |  |
| **UHDRS TFC** | 0.32 (0.12) | 0.25 (0.14) | **0.28 (0.14)** | **0.28 (0.14)** | 0.27 (0.15) | 0.23 (0.12) | -0.08 (0.12) | 0.09 (0.10) |  |
| **STROOP WORD** | 0.57 (0.13) | **0.37 (0.15)** | 0.37 (0.16) | 0.37 (0.17) | 0.36 (0.18) | 0.29 (0.15) | -0.09 (0.14) | 0.01 (0.19) |  |
| **SDMT** | 0.46 (0.17) | **0.28 (0.17)** | 0.27 (0.18) | 0.27 (0.18) | 0.26 (0.17) | 0.25 (0.16) | -0.11 (0.19) | -0.04 (0.21) |  |

**Table S6**. Model performance in terms of average R² (SD) across 50 cross-validation folds for different sets of demographic features. Column 1 shows the performance of our automated model for comparison. CAP score alone achieved the highest R² for cognitive scores, while both combinations (CAP score + CAG repeat length and CAP score + Age) achieved the highest R² for TMS and cUHDRS.


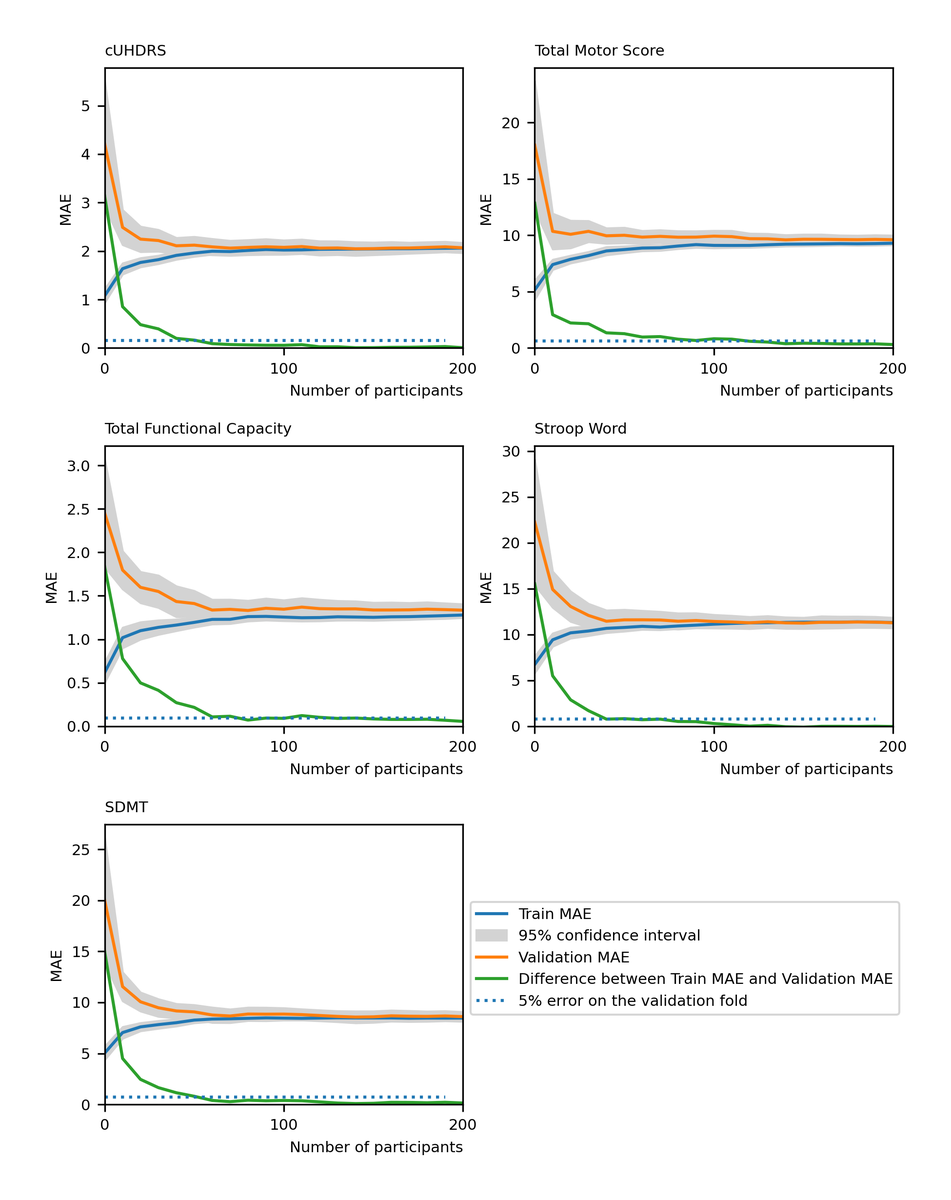


**Figure S2**. Estimating the minimum sample size for model calibration. To assess the sample size required for convergence between training and validation MAE, we simulated 30 random shuffles of participant order. For each shuffle, sample size was increased by 10 participants at each step (80% training, 20% validation), and the MAE was computed. Train and validation MAE represented lines are mean over these 30 simulations with their 95% confidence interval. The green line shows the MAE difference between validation and training sets. The blue dotted line marks a 5% residual error threshold (5% of initial validation MAE), considered acceptable for convergence. Convergence was reached at N=100 for all scores, and at N=50 for TMS and Stroop Word, providing practical guidance for future multilingual studies.


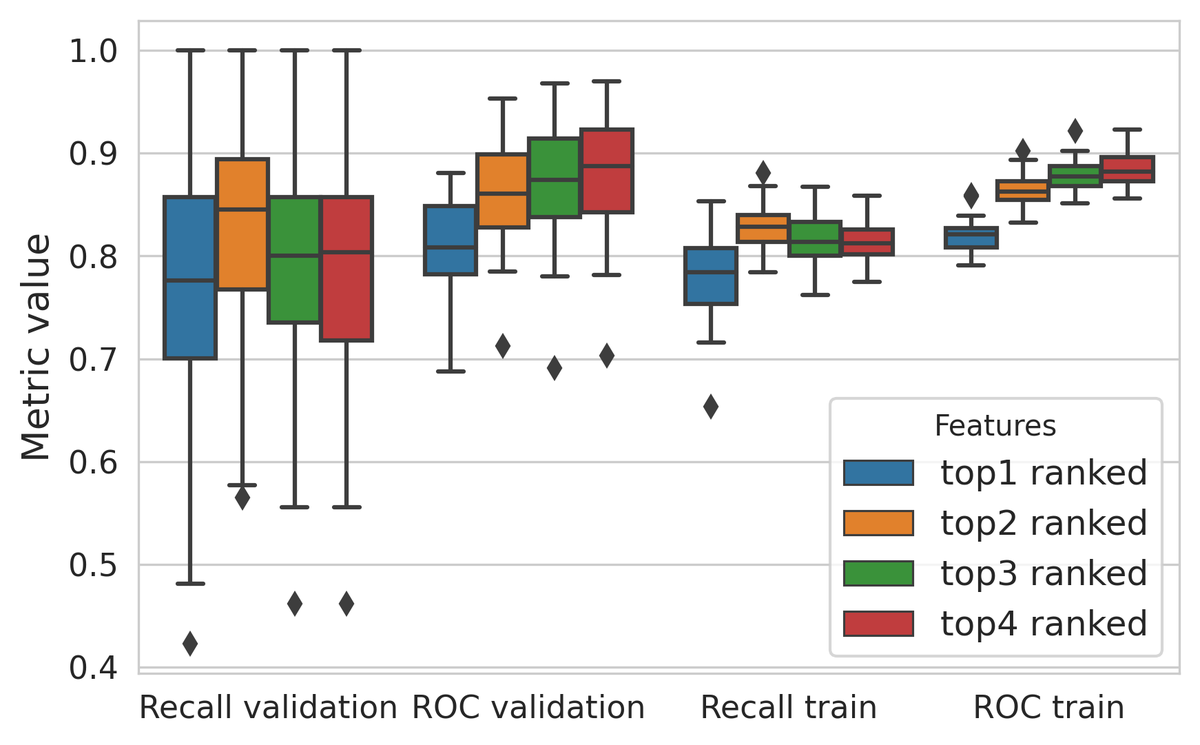


**Figure S3**. Cross-validation Performance of logistic regression models for classifying HD-ISS Stage 3 vs. Stages 0-2 on validation and train folds. We tested combinations of the top four features identified in the initial regression model. Performance (ROC AUC and recall) was averaged over 50-fold cross-validation (80% training, 20% validation). As shown, using the top two features (CAP score and speech rate) improved performance over CAP score alone, with minimal gains from additional feature for ROC AUC. These two were retained in the final model for simplicity and interpretability.


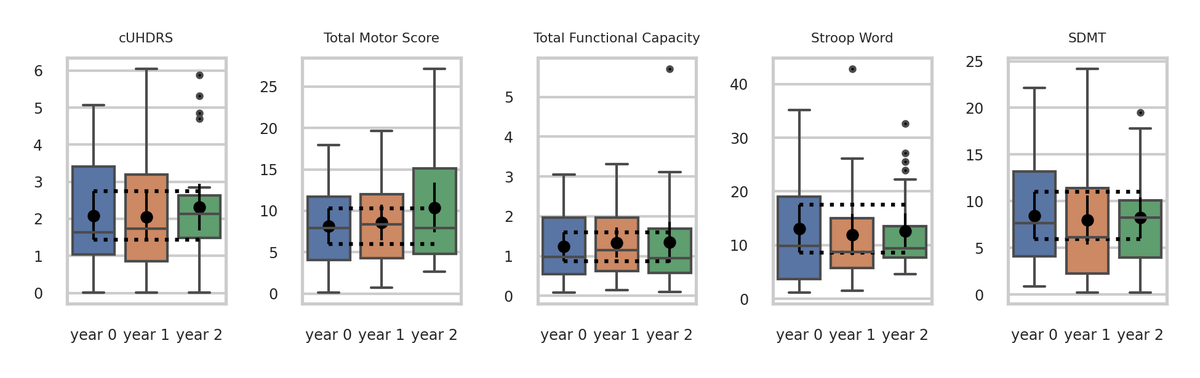


**Figure S4**. Boxplots of Absolute error per visit and per clinical score. Boxplot components: center lines indicate medians; boxes span the interquartile range (IQR); whiskers extend to 1.5 × IQ. The MAE per visit is shown as a dot, with a bold line indicating its 95% confidence interval. The 95% confidence interval from the first visit’s MAE is shown as a horizontal dotted line for cross-visit comparison. All visit-specific means fall within this interval, except for TMS at Year 2.


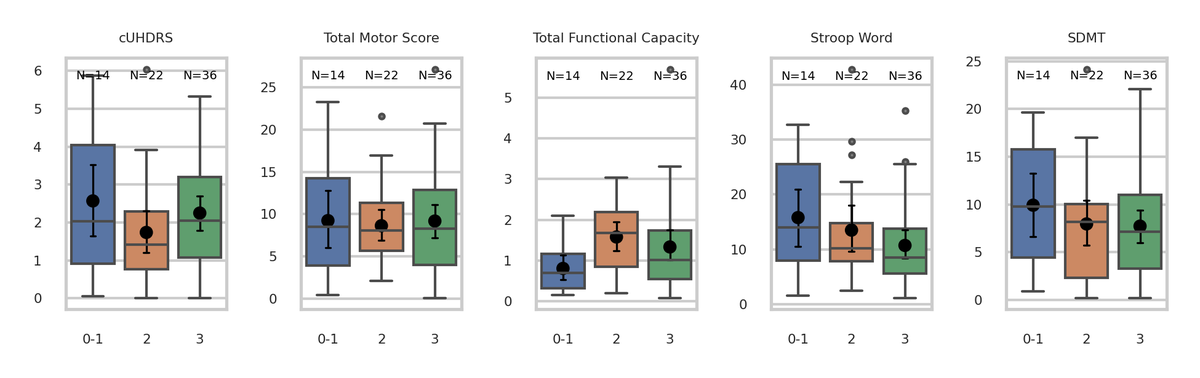


**Figure S5**. Boxplots of Absolute Error by HD-ISS Stage and clinical score on the Test Set (24 participants). The HD-ISS Stage was defined at the visit level. The MAE per stage is shown as a dot, with a bold line indicating its 95% confidence interval. Boxplot components: center lines indicate medians; boxes span the interquartile range (IQR); whiskers extend to 1.5 × IQR. Overall trends: cUHDRS MAE decreased from 2.6 (14.7% in relative terms, see Method’s definition) at Stage 0-1 to 1.7 (13.4%) at Stage 2, then increased to 2.2 (24%) at Stage 3. Cognitive scores showed decreasing absolute MAE with disease stage: SDMT MAE dropped from 9.9 (18%) at Stage 0-1 to 7.7 (29%) at Stage 3; SW MAE similarly decreased from 15.8 (15.3%) to 10.7 (17.6%). TMS showed higher relative errors, with MAE of 8.6 (43.4%) at Stage 2 and 9.2 (30%) at Stage 3.


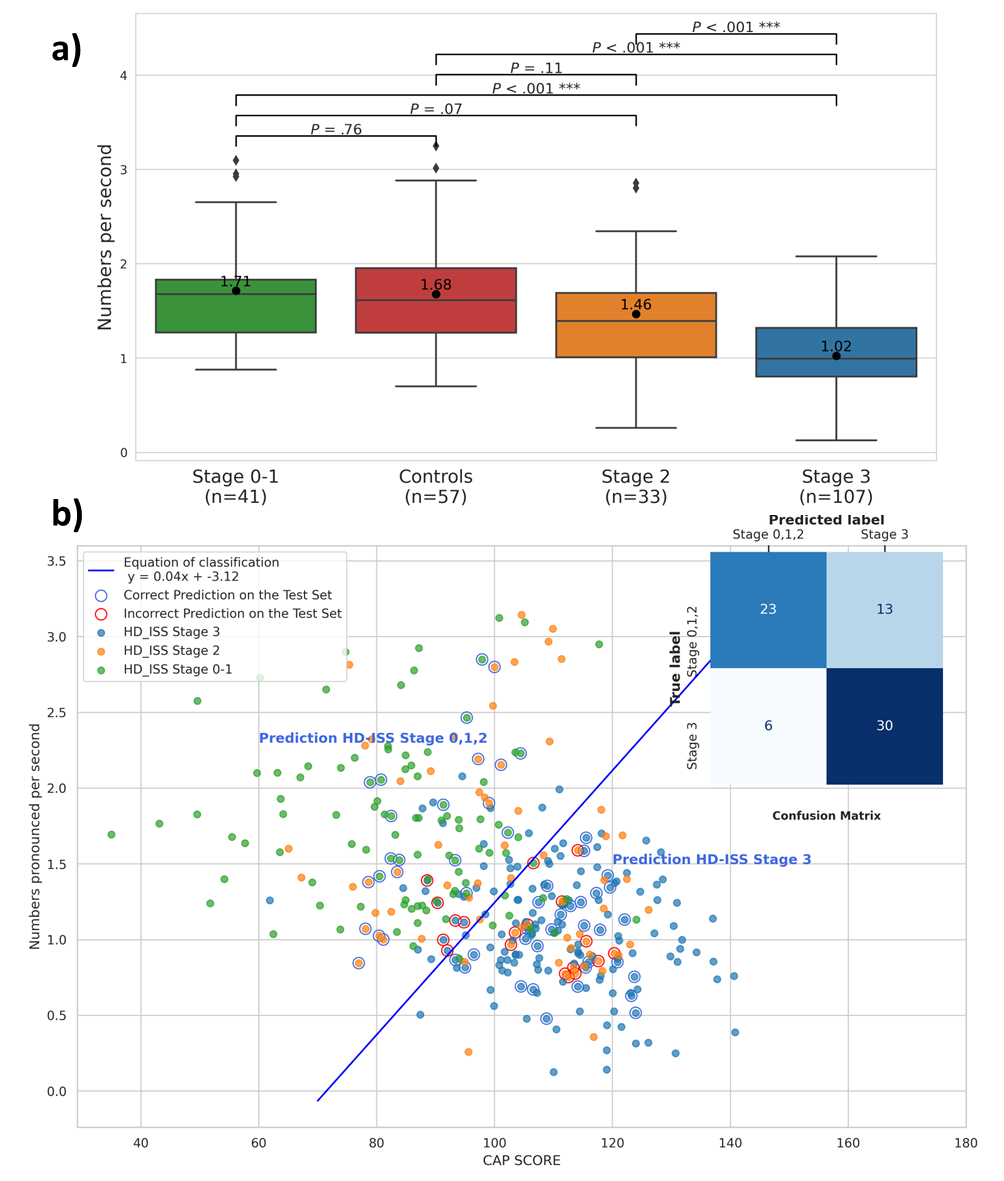


**Figure S6**. Speech rate as a biomarker of HD-ISS Stage 3. 5.a) Boxplots of speech rate (numbers pronounced per second) across HD-ISS stages, based on the first visit of the full dataset. Control participants from the BIO-HD study are shown for comparison to replicate findings from a previous study⁹. Speech rate was significantly reduced in participants at Stage 3 compared to earlier stages (one-way ANOVA followed by post hoc Tukey test, P <.001). 5.b) Scatterplot of speech rate versus CAP score, with logistic regression-based classification of HD-ISS Stage 3 (vs Stages 0-2). The blue line indicates the decision boundary. Each point represents a visit; test set samples are shown as circles (blue for correct classification, red for incorrect). Color shading indicates true HD-ISS stage. A confusion matrix summarizing model performance on the test set is shown in the top-right panel (ROC AUC = 0.79).

| Score | Factor | F(df1,df2) | p | np² | Post hoc (Time) |
| --- | --- | --- | --- | --- | --- |
| cUHDRS | Time | 17.83(2,46) | P<.001 | 0.02 | Y1 vs Y2, Y1 vs Y0, Y2 vs Y0 |
|  | Type | 0.17(1,23) | .68 | 0.00 |  |
|  | Time * Type | 2.13(2,46) | .13 | 0.00 |  |
| UHDRS TMS | Time | 8.76(2,46) | .001 | 0.01 | Y1 vs Y2, Y2 vs Y0 |
|  | Type | 0.01(1,23) | .93 | 0.00 |  |
|  | Time * Type | 0.39(2,46) | .68 | 0.00 |  |
| UHDRS TFC | Time | 8.85(2,46) | .001 | 0.02 | Y1 vs Y0, Y2 vs Y0 |
|  | Type | 2.27(1,23) | .15 | 0.02 |  |
|  | Time * Type | 2.14(2,46) | .13 | 0.01 |  |
| SW | Time | 14.23(2,46) | P<.001 | 0.02 | Y1 vs Y2, Y1 vs Y0, Y2 vs Y0 |
|  | Type | 0.0(1,23) | .98 | 0.00 |  |
|  | Time * Type | 3.16(2,46) | .05 | 0.00 |  |
| SDMT | Time | 9.94(2,46) | P<.001 | 0.01 | Y2 vs Y0 |
|  | Type | 0.02(1,23) | .88 | 0.00 |  |
|  | Time * Type | 0.54(2,46) | .59 | 0.00 |  |

**Table S7**. Results of repeated-measures two-way ANOVA with factors Time (Y0, Y1, Y2) and Type (clinical vs xHD-Vox). Time had a significant effect on all scores, with no significant Time × Type interactions. Post hoc Tukey tests were conducted for pairwise comparisons across Time.


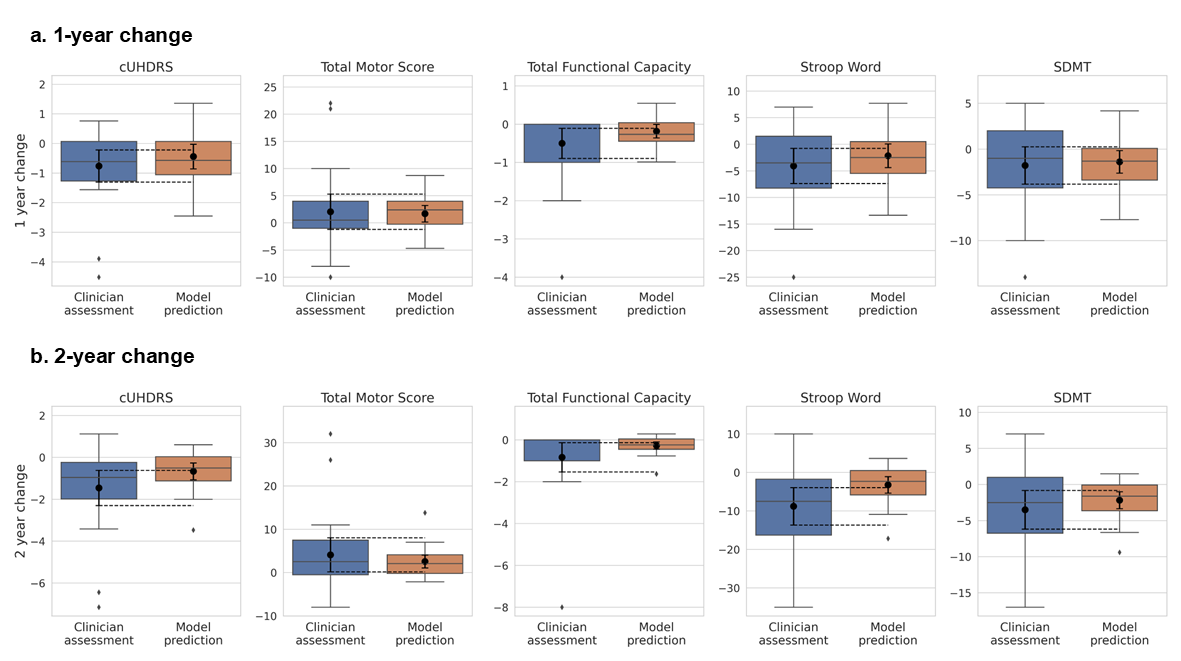


**Figure S7**. Model evaluation for disease progression tracking using longitudinal data on the test set (N=24 participants, 3 annual visits each). Panels (a) and (b) show boxplots of one-year and two-year changes in clinical scores, comparing clinician assessments with model predictions.
One-year change is defined as Score at Year 1 - Score at Year 0; two-year change as Score at Year 2 - Score at Year 0. The magnitude of decline at the population level is indicated by the mean change (shown as a dot), with a bold line representing its 95% confidence interval. A horizontal dotted line indicates the 95% confidence interval of the clinician assessments, provided as a reference for comparison with model predictions. Detailed mean change and 95% CIs are provided in Table 6 of the main manuscript. Boxplot components: center lines indicate medians; boxes span the interquartile range (IQR); whiskers extend to 1.5 × IQR.


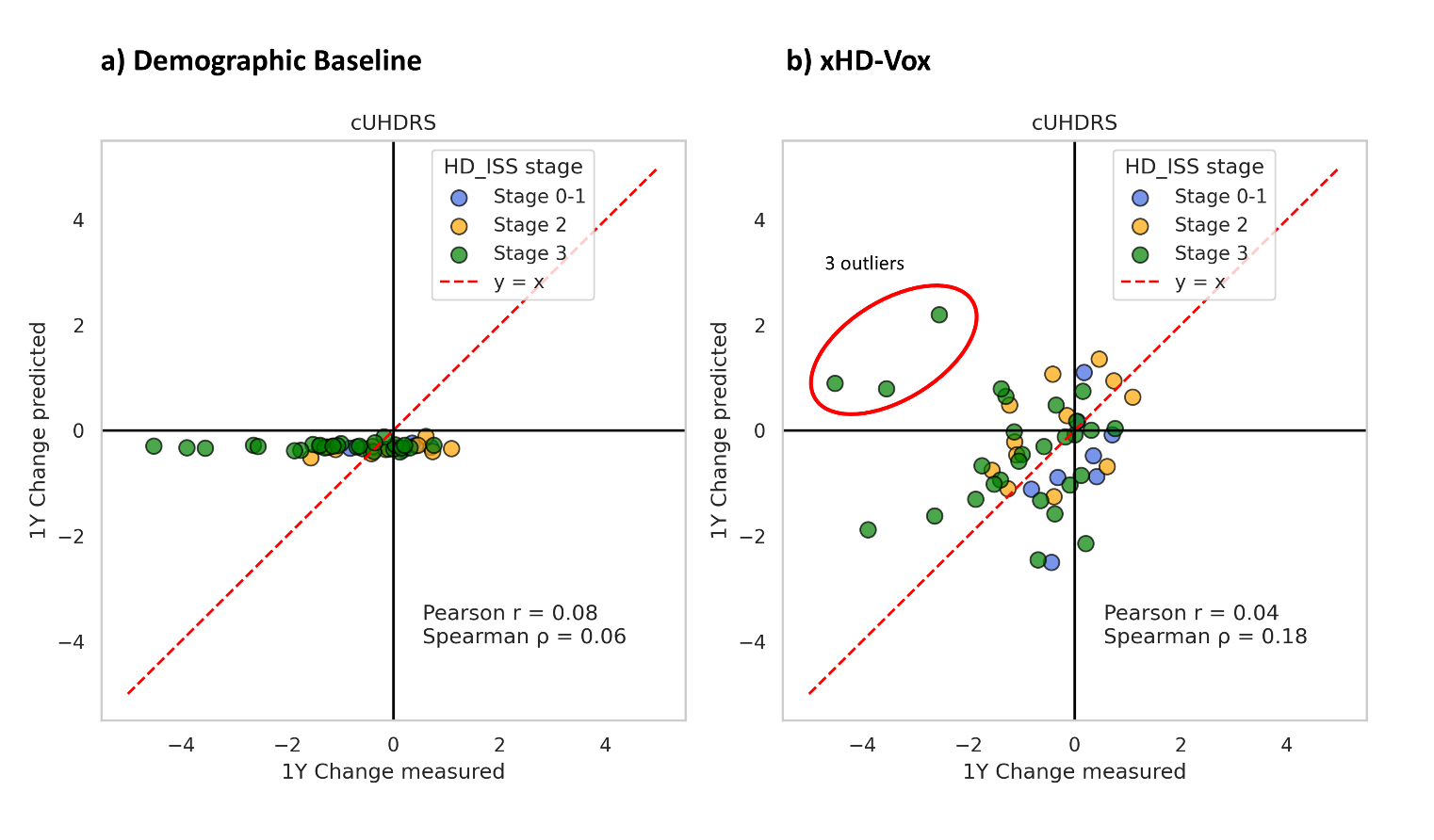


**Figure S8**. One-year change in cUHDRS predicted versus clinically measured for xHD-Vox (panel a) and the demographic baseline model (panel b) without 3 outliers. The red line represents the line of perfect agreement (y = x). Pearson and Spearman correlation coefficients are reported.


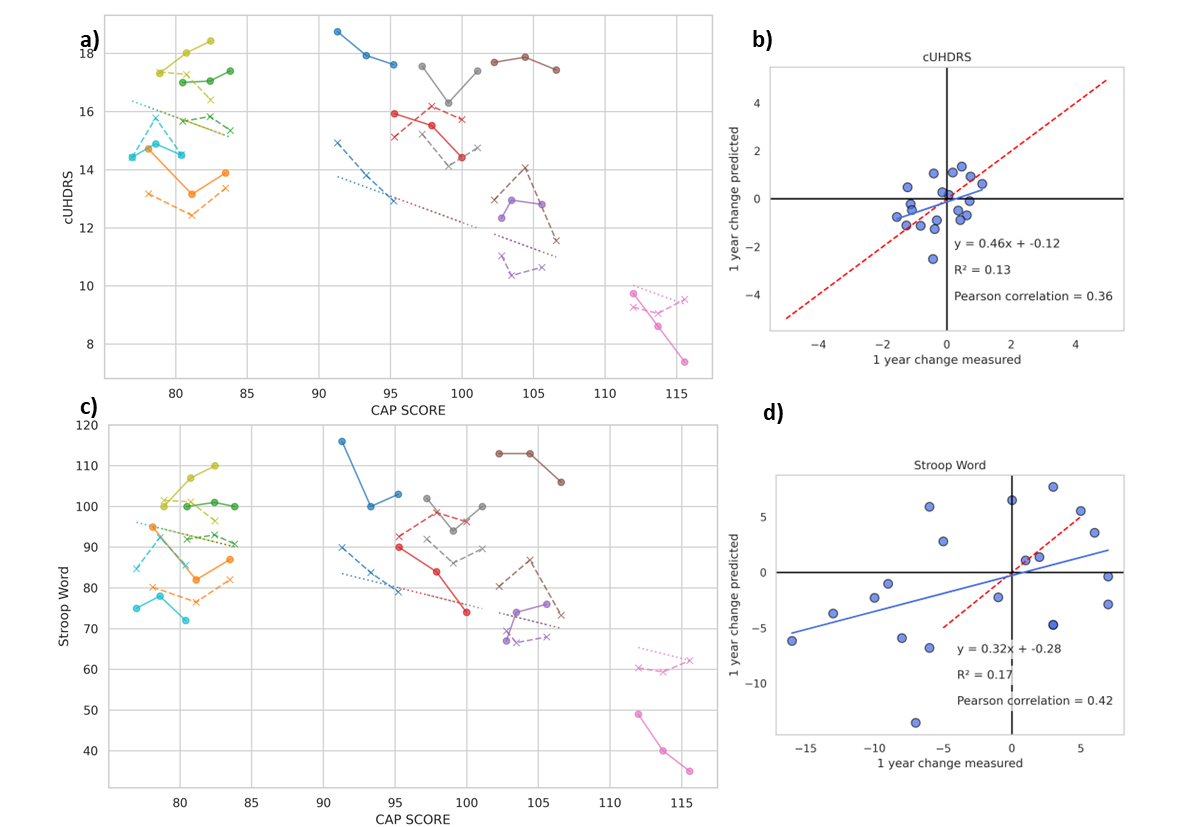


**Figure S9**. Longitudinal evolution of participants in HD-ISS stage 0-1 or 2 of the Test set. Figures a and c display individual trajectories over the two-year follow-up period for cUHDRS and Stroop Word performance, respectively, as a function of CAP score. The solid lines represent the measured trajectories for each participant, while the dashed lines indicate trajectories predicted by our model. These lines connect annual visits, with circles indicating measured clinical scores and crosses representing model predictions. The dotted line shows the predicted trajectory based solely on the CAP score. Each participant is identified by a unique colour. Figures b and d present the same information as Figures a and c, respectively, but in a different format: scatter plots comparing the predicted and measured one-year change in the clinical measure. The red line represents the line of perfect agreement (y = x), while the blue line indicates the linear regression between predicted and measured one-year changes. The regression equation and R² value are shown in the legend. Pearson correlation coefficients are also reported.
